# Sex Steroid Metabolism in Benign and Malignant Intact Prostate Biopsies: Individual Profiling of Prostate Intracrinology

**DOI:** 10.1155/2014/464869

**Published:** 2014-08-13

**Authors:** Daniele Gianfrilli, Silvia Pierotti, Riccardo Pofi, Costantino Leonardo, Mauro Ciccariello, Federica Barbagallo

**Affiliations:** ^1^Department of Experimental Medicine, Sapienza University, Viale del Policlinico 155A, 00161 Rome, Italy; ^2^Department of Urology, Sapienza University, 00161 Rome, Italy; ^3^Department of Radiology, Sapienza University, 00161 Rome, Italy

## Abstract

*In vitro* studies reveal that androgens, oestrogens, and their metabolites play a crucial role in prostate homeostasis. Most of the studies evaluated intraprostatic hormone metabolism using cell lines or preprocessed specimens. Using an *ex vivo* model of intact tissue cultures with preserved architecture, we characterized the enzymatic profile of biopsies from patients with benign prostatic hyperplasia (BPH) or cancer (PC), focusing on 17*β*-hydroxy-steroid-dehydrogenases (17*β*-HSDs) and aromatase activities. Samples from 26 men who underwent prostate needle core biopsies (BPH *n* = 14; PC *n* = 12) were incubated with radiolabeled ^3^H-testosterone or ^3^H-androstenedione. Conversion was evaluated by TLC separation and beta-scanning of extracted supernatants. We identified three major patterns of conversion. The majority of BPHs revealed no active testosterone/oestradiol conversion as opposed to prostate cancer. Conversion correlated with histology and PSA, but not circulating hormones. Highest Gleason scores had a higher androstenedion-to-testosterone conversion and expression of 17*β*-HSD-isoenzymes-3/5. *Conclusions*. We developed an easy tool to profile individual intraprostatic enzymatic activity by characterizing conversion pathways in an intact tissue environment. In fresh biopsies we found that 17*β*-HSD-isoenzymes and aromatase activities correlate with biological behaviour allowing for morphofunctional phenotyping of pathology specimens and clinical monitoring of novel enzyme-targeting drugs.

## 1. Introduction

Prostate cancer is the most common cancer in men. An increasing trend in prostate cancer incidence, a disease associated with age, has been described and partially attributed to better screening procedures [1] and awareness [2]. A significant number of prostate cancers, however, remain indolent and, if untreated, do not alter life quality and expectancy. For this reason the burden of universal treatment of confined asymptomatic disease should be weighed against the economic socioeconomic costs of overtreatment, the complications associated with the currently available treatments (including androgen-deprivation therapy), and the overall quality/life expectancy of affected subjects [3, 4].

Improvements in prostate cancer diagnosis, classification, and treatment witnessed in the past 20 years have not been paralleled by improvement in preoperative prognostic grading of the disease which still relies on morphological appearance of random biopsies. A functional prognostic presurgical characterization of the disease is needed to identify those subjects who require aggressive treatment and those who can be managed conservatively. Furthermore, a function profiling of prostate tissue will also be very useful to monitor unoperated patients during radiotherapy (RT) and androgen-deprivation therapy (ADT) to follow up changes in prostate tissue responsiveness and aggressiveness.

It is widely accepted that androgens play a central role in the biology of the prostate. Estrogens, however, can also modulate prostatic growth and development [5, 6]. Taken together, observations from many studies on murine models imply that both androgens and estrogens are needed to induce proliferative, precancerous lesions and prostate cancer. Indeed, the balance between androgen induced cell proliferation and apoptosis is thought to be a major regulator of growth of the normal and cancerous prostate. Epidemiological studies, however, showed that there is no association between circulating steroid hormone levels and prostate cancer [7].* In vitro* studies reveal that intratissual levels of sex steroids may diverge from their plasmatic counterpart due to complex enzymatic equipment expressed by prostate cells that can interconvert steroids [8]. Labrie et al. was the first to describe the “intracrinology” of the prostate gland [9, 10].

Prostate tissue contains a variety of steroid metabolizing enzymes required for the local production of active androgens and estrogens from their precursors provided by the adrenals [10–12]. The main enzymes involved in local steroid metabolism are steroid sulfatases, 3*β*-hydroxysteroid dehydrogenases (3*β*-HSDs), 17*β*-hydroxysteroid dehydrogenases (17*β*-HSDs), 5*β*-reductases, and aromatase. In normal conditions a steady state exists between synthesis and inactivation of active androgens; however tissue transformation can be associated with an alteration of this balance. Increasing evidences suggest that prostate cancer cells alter local and paracrine steroid hormone metabolism. In the past decade, a growing number of studies tried to explore the role of local androgen production in cancer progression and transformation into castrate-resistant tumours (CRPC) [13, 14].

In the present work we provide evidences that intraprostatic hormonal profiling, some sort of individual metabolic fingerprint, can be easily obtained. One of the most innovative features of the present study is that we analysed the metabolism of prostate cells directly “*ex vivo*” on fresh specimens from biopsy or surgical resection. The aim of the current work is (1) to set up a reproducible, rapid, and easy approach to define the enzymatic profiling of the normal, hyperplastic (BPH), and cancerous prostate cell (PC) (2) to correlate the patterns with steroid enzymes' expression and tumour's histology.

## 2. Methods

### 2.1. Patients and Tissues

Specimens were obtained from 26 patients (14 BPH and 12 PC) who underwent transrectal ultrasound-guided prostate biopsy followed by prostate surgery (radical for cancer or transurethral for enlargement) between 2009 and 2012 at the Department of Urology, Sapienza University. The clinical characteristics are reported in Table 1.

All patients examined in this study did not receive radiation, chemotherapy, or hormone therapy before surgery. Clinical data, including patient age, serum prostate specific antigen (PSA) concentration, clinical stage according to the International TNM classification, lymph node status, and Gleason's score, were retrieved for all patients. All procedures were performed using commercially available ultrasound equipment with 7.5 MHz probes (Philips IU22); biopsy samples were obtained using an automatic spring-loaded biopsy gun with an 18-gauge needle.

**Figure 1:**
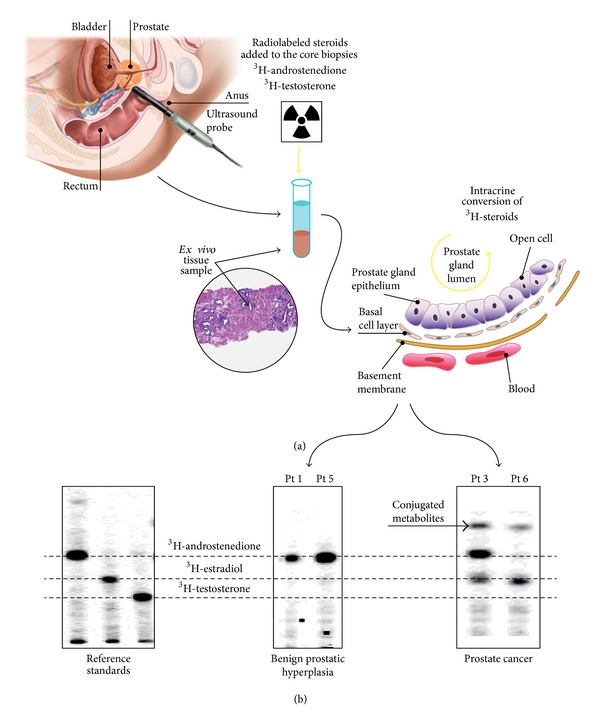
(a) Unique features of our “*ex vivo*” model: samples were histologically confirmed on-site, part of each sample was immediately dry frozen for subsequent RNA analysis, and part was fixed and embedded for paraffin section analysis. The core biopsies guaranteed tissue integrity that is crucial to maintain enzyme kinetics and directionality closer to what occurs in *in vivo*; specifically the stromal-to-epithelial interaction remains unaltered, allowing appraisal of the contribution of tumor microenvironment. (b) Examples of different TCL patterns (steroid fingerprint) in patients with BPH or PC (monodimensional development). Of notice is that patients with different stage PC exhibit different steroid maps (patient 3 had a Gleason 6, while patient 6 a Gleason 8). Incubation time: 24 hours. BPH: benign prostatic hyperplasia; PC: prostate cancer.

Specimens upon collection were placed on saline buffer and immediately processed (Figure 1). The protocol was reviewed and approved by the local board and funded by study Grants MIUR 2008NY72SJ and RBFR10URHP.

### 2.2. Enzymatic Assays

Unprocessed samples were split into three parts, one for enzymatic activity, one for mRNA gene expression studies, and one sent to the pathologist for confirmation. One hundred milligrams of intact tissue was exposed to physiological concentrations of different H_3_-labeled compounds (Sigma-Aldrich), to explore the capability of these cells to metabolize these substances, in serum-free buffering medium under controlled temperature and atmosphere. One hundred microliters of media was collected at different time points (30 min, 1 h, 2 h, 4 h, 8 h, and 16 h) after incubation of various H_3_-labeled steroids and total lipids were extracted with 400 *μ*L Folch reagent (chloroform/methanol: 2 : 1 vol/vol), vortexed, and spun at 14,000 ×g for 5 min. The organic phase was collected and evaporated in a speed-vac. Dried extracts were redissolved in 40 *μ*L of ethanol and spotted onto TLC plates (Whatman). The plates were developed twice in chloroform-ethyl acetate (4 : 1 vol/vol). Steroid metabolites were quantified using a BioScan AR-2000 Imaging System (Bioscan). Bidimensional acquisition of *β*-emission was obtained using dedicated software (Bioscan).

### 2.3. Reverse Transcriptase-Polymerase Chain Reaction (RT-PCR)

RNA was extracted from tissue samples using GenElute TM Mammalian Total RNA Miniprep kit according to the manufacturer's instructions (Sigma-Aldrich). 1 *μ*g of total RNA was retrotranscribed in a total volume of 50 *μ*L using random primers (F. Hoffmann-La Roche, Basel, Switzerland) and M-MLV reverse transcriptase (Invitrogen, Carlsbad, CA) and used as template for real-time polymerase chain reaction. Real-time quantitative PCRs (qPCR) was performed using 2 *μ*L of cDNA, 18 *μ*M for each primer, 5 *μ*M for probe (Applied Biosystems), TaqMan GenEX Master Mix (Applied Biosystems), and iQCycler (Bio-Rad Laboratories) according to the manufacturer's instructions. 17*β*-HSD1, 17*β*-HSD2, 17*β*-HSD3, 17*β*-HSD4, 17*β*-HSD5, 17*β*-HSD7, 17*β*-HSD8, 17*β*-HSD10, aromatase, and beta-actin (actb) human primers were used. Data were analyzed using the standard curve method. The relative quantities of transcripts were calculated from triplicate samples after normalization of the data against the housekeeping gene (actb).

### 2.4. Data Analysis and Statistical Methods

Differences between experimental groups were analyzed by the Student's *t*-test and chi-square test. Spearman's correlation coefficients were used to assess the relationship between experimental variables. Multiple comparisons were performed using a one-way ANOVA and Turkey's post hoc test. The test was two-sided and *P* ≤ 0.05 was considered significant. All analyses were performed using SPSS version 17.0 PC version (SPSS Inc., Chicago, IL, USA).

## 3. Results

Clinical features of the enrolled patients are reported in Table 1. None of the recruited patients were taking steroid hormones or chemotherapy or received previous external beam radiation. Two PBH patients were on *α*-adrenolytic treatment of lower urinary tract symptoms.

To determine the efficiency whereby various precursors undergo intraprostatic conversion to more potent steroids, an operation protocol has been designed as follows: normal and cancerous cells derived from bioptic specimens were exposed to physiological concentrations of different labelled compounds (herein described for ^3^H-androstenedione or ^3^H-testosterone) as described in methods. Enzymatic products were then separated by two-dimensional TLC (2D-TLC) by sequential use of two customized mobile phases in order to discriminate molecular compounds that may differ for a single atom of hydrogen. The 2D procedure started with a classical monodimensional TLC (as shown in Figure 1) that, when necessary, could be followed by a further separation based on the affinity with the second mobile phase run orthogonally to the first one. Because it is unlikely that two molecules will be similar in two distinct repartition properties, molecules are more effectively separated in 2D-TLC than in 1D-TLC. As a result the products of hormone metabolism spread out across the whole chromatographic surface. In respect to the current analysis of androstenedione/testosterone conversion into estrogens or testosterone a 1D-TLC was sufficient to discriminate the various steroids. Subsequent scanning of the developed TLC sheet, by means of the Argon-Methane enhanced *β*-emission scanner, will allow the identification of substrate-product(s) emitting spots and the measurement of CPM from each spot. Bioscan software renders an image that is equivalent to a “metabolic fingerprint” of each prostatic specimen (Figure 1(b)).

**Figure 2:**
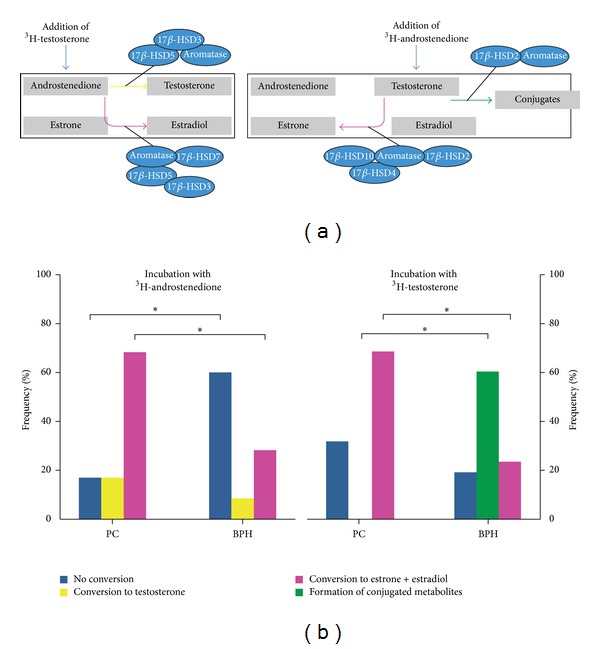
(a) Schematic representation of the different pathways evaluated by TLC byproducts formation after exposure to ^3^H-androstenedione or ^3^H-testosterone. (b) Frequency of the major pattern of conversions observed during incubation in all subjects (more than one conversion reaction can be observed in the same subject). *P < 0.05; BPH: benign prostatic hyperplasia (*n* = 14); PC: prostate cancer (*n* = 12).

A representative pattern of conversion that has been observed in BPH and in PC is shown in Figure 1(b). When PC samples were exposed to ^3^H-androstenedione the most frequent observed pattern was estradiol conversion (66%), while 17% of tissue revealed testosterone formation and the remaining 17% showed no conversion activity (Figure 2). On the contrary the majority of BPH samples showed no conversion (60%) or estrogen formation (30%); only 10% of samples exhibited some testosterone production. When PC samples were exposed to ^3^H-testosterone, the majority (66%) revealed estrogen formation or no conversion (34%); none showed formation of conjugated byproducts. On the contrary in BPH samples 60% of subjects showed formation of conjugated steroids, while estrogen formation was observed in 22% and 18% exhibited no conversion (Figure 2).

**Figure 3:**
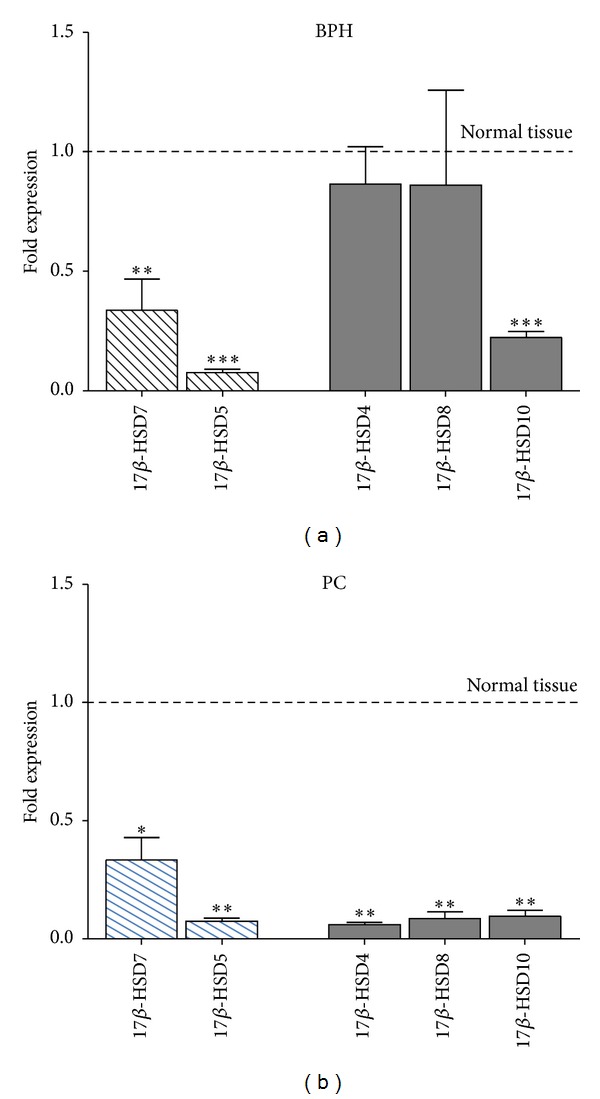
Quantitative gene expression in examined tissue relative to whole (normal) prostate tissue (fold change) of 17*β*-HSDs with predominant reductase activity (types 5 and 7, yellow) versus 17*β*-HSDs with a predominant oxidase activity (types 4, 8, and 10, green). BPH: benign prostatic hyperplasia (*n* = 11); PC: prostate cancer (*n* = 7). Statistical significance is represented versus whole normal tissue ***P ≤ 0.001; **P < 0.01.

The synthesis of testosterone from precursor molecules occurs via a well-established sequence of reversible reactions. Since androgen levels may be affected by both changes in synthetic and degradative enzyme expression, gene expression analyses of 17*β*-HSD types 5 and 7 isoenzymes with predominant reductase activity versus types 4, 8, and 10 with a predominant oxidase activity were performed in BPH and PC (Figure 3). Compared to normal tissue, BPH showed a significant lower expression level of the 17*β*-HSD types 5, 7, and 10 enzymes, consistently with a reduced de novo enzymatic production of androgens; in respect to PC, all samples showed lower levels of expression for all enzymes, compared to BPH tissue. This finding is in apparent contrast with the enzymatic conversion activities reported in Figure 2. Indeed, PC showed a predominant reduction in the expression of the 17*β*-HSDs with reductase activity compared to oxidizing ones, when compared with BPH or normal tissue.

**Figure 4:**
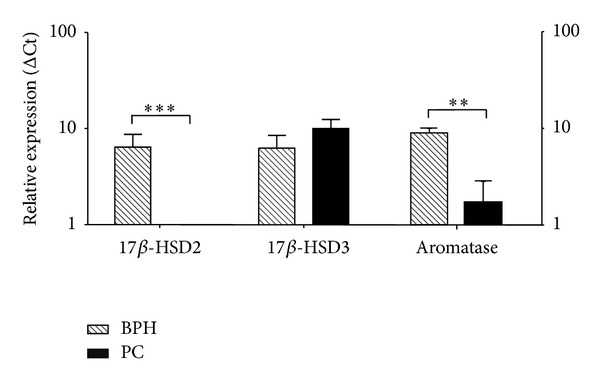
Quantitative gene expression in examined tissue relative to whole (normal) prostate tissue (normal set to 1) of 17*β*-HSD type 2 and type 3 and aromatase. BPH: benign prostatic hyperplasia (*n* = 11); PC: prostate cancer (*n* = 7).

Moreover none of the PC samples expressed 17*β*-HSD type 2, compared to BPH, while maintaining an efficient expression of 17*β*-HSD type 3 enzymes and aromatase transcripts (Figure 4).

**Figure 5:**
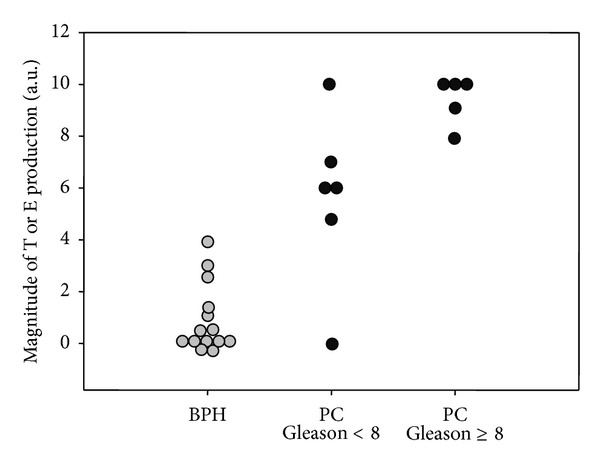
Correlation between conversion efficiency toward testosterone or estradiol and histological grading of tissue samples; BPH: benign prostatic hyperplasia (gray circle, *n* = 14); PC: prostate cancer (solid circle, *n* = 12).

In Figure 5 estradiol and testosterone generation in all samples (compared to the maximum conversion achieved set to 10) is reported. Despite the relatively small cohort number, a greater enzymatic activity is seen in PC samples with a high grade Gleason score compared to low Gleason score (histology) (*P* < 0.05).

## 4. Discussion

Prostate cancer is considered a “hormone-dependent” disease because the prostate requires testicular androgens for its secretory function and cancerous cells retain this sensitivity to androgens. Cell growth and survival of early stage prostate cancer can in fact respond to androgens and this evidence is the background of the androgen-deprivation therapy (ADT). Although plasma concentrations of testosterone have been shown to decrease by more than 90% following castration, androgen levels in prostate cancer tissues decreased only by 50–60%, suggesting the importance of in situ androgen production in prostate cancers [6, 15, 16]. In addition, ADT is associated with severe systemic adverse events such as cardiac, metabolic, hepatic, bone, sexual, and cognitive complications that eventually lead to an increased mortality rate compared to the risk related to prostatic cancer itself [3, 4]. ADT is successful until the tumour enters an androgen-refractory state, leading to the failure of such long-term strategy.

So far it has been impossible to associate high levels of circulating androgens with the progression of prostate cancer [7]. This is in strong agreement with the decline of plasmatic testosterone with age, which would ultimately lead to an inverse relationship between circulating androgens and the risk of developing prostate cancer. As a matter of fact, intratissual levels of sex steroids may diverge from their plasmatic counterpart due to enzymatic equipment that can interconvert and metabolize steroids [9, 10, 15]. The local steroid metabolism is therefore the main determinant of the intraprostatic hormonal profile. Androgens level variation as sole determinants of prostate cancer development and progression has led to neglecting the evidence that the onset of malignancy is accompanied by an estrogen-sensitive condition where tumour growth and spread is stimulated and maintained by an increase in the cellular levels of androgen aromatization into estrogens, in a manner similar to that described in breast cancer [6, 11]. Moreover, animal models have clearly showed that supraphysiological levels of estrogens and androgens are each separately capable of altering the normal growth of the prostate, but individually they do not induce prostatic malignancy. As neither hormone by itself is able to induce malignant changes in the prostate, the balance between sex steroids is critical in inducing premalignant and malignant lesions. In this respect, an altered profile of prostatic enzymes that metabolize steroids has to be invoked in the acquisition of aggressiveness of prostate cancer. At present, we barely know the exact molecular mechanisms underlining the progression of the prostatic disease and the acquisition of the metastatic behaviour.

Classical monitoring factors of prostate cancer, such as PSA levels, stadiation, and Gleason score, are losing their reliability in the attempt to discriminate among multiple stages of the disease. New prognostic and diagnostic markers are needed. Our goal was to develop novel methods to acquire data on the enzymatic profile (and its changes) in intact prostate tissue in order to characterize the history of the disease with respect to follow-up, medical, surgical, and radiotherapeutic procedures.

In addition, there is a recent growing interest toward several metabolites that could be bioactivated into more active steroids with high affinity binding to androgen receptor (AR) or estrogen receptor (ER) [17]. An interesting observation is that androgenic activity of the C11-keto forms of A4, T, and DHT are more androgenic than their respective 11-hydroxy forms [17]. This implicates that the activity of the 11*β*-HSDs family, which interconvert 11-hydroxy and 11-keto steroids, could be another regulatory point in the activation or inactivation of 11-hydroxy and 11-keto androgens in the prostate [17]. The involvement of 11b-HSD in prostate physiology deserves future studies as it has already been shown to be crucial in several clinical conditions [18, 19].

The most extensively studied intracrinology pathways, nowadays, are those related to the 17*β*-HSDs.

Precise measurements of relative enzymatic activity of this family are complicated by the requirement for optimal pH and nicotinamide adenine dinucleotide cofactors that requires intact cell models. In this respect we believe that our model has the advantage of assessing this in the best way. In androgen and estrogen metabolism, 17HSDs catalyze the reactions between the active 17*β*-hydroxysteroids and less active 17-ketosteroids. At present, several 17HSD isoenzymes have been characterized [20]; specifically types 1, 3, 5, and 7 are reductive enzymes, whereas types 2, 4, 8, 10, and 11 are oxidative enzymes. Very recently Fankhauser and colleagues showed that upregulation of 17HSD17B is the predominant source of signalling androgens in hormone refractory prostate cancer, much greater than either the so-called “backdoor” or the “5-*α* dione” pathway [14].

Our findings document that in an* ex vivo* model of intact prostate tissue, an enhanced reductive pathway is a feature of PC, compared to BPH. These data are consistent with those by Nakamura et al., demonstrating that in human prostate cancer 17*β*-HSD5 immunoreactivity was detectable in 77% of cases with a stronger staining correlated to more advanced clinical stages (TNM stage pT3 versus pT2) [21].

The current idea is that the progression of prostate cancer in the setting of castrate androgen levels is not due to the development of an androgen insensitive tumour clone but rather to the fact that the cancer has evolved mechanisms to escape systemic androgen deprivation while still taking advantage of signalling through AR [6, 13–15, 22].

The recognition of the fact that intratumoural androgen synthesis and activity are biologically relevant and that overexpression of the AR is a consistent feature of prostate cancer progression has led to the development of several new therapeutic approaches. One example is the utilization of abiraterone acetate, an inhibitor of 17*α*-hydroxylase and C17, 20 lyase (CYP17A1) for advanced CRPC treatment. Blockade of CYP17A1 activity by abiraterone suppresses androstenedione, dehydroepiandrosterone (DHEA), testosterone, and oestradiol formation, as well as other metabolites [23]. We believe that our approach could be useful to monitor intracrinology changes of patients under novel enzyme-targeting drugs.

In the innovation of the methodological approach, we rely on several facts: (1) because of the high sensitivity of the detection system, the enzymatic activity can be determined using exceedingly low amounts of labelled compounds mimicking the* in vivo* conditions; (2) the number of cells required to test the activity is also very small allowing test on biopsies; (3) enzymatic activity is assessed in the optimal pH and nicotinamide adenine dinucleotide cofactors concentration; (4) tissues can be subsequently processed for other uses.

The present work, however, has also some limitations. One limitation is that we did not measure DHT levels that could have been altered by both type 1 and type 2 5-*α* reductase activity [21]. However, taking into account that patients with BPH or prostate cancer often use specific 5-*α* inhibitors (2 out of 14 in our series) this was not possible. Another limitation of the present study is that we did not address the role 17*β*-HSD6. This enzyme has been recently considered the backdoor pathway of DHT synthesis in patients undergoing ADT [24] since it exerts a key role in androstanediol bioactivation to the downstream pathway leading to DHT formation from adrenal steroids [25]. However,* in vitro* assessment of 17*β*-HSD6 activity is very problematic and most of studies simply rely only on mRNA expression, that, as we have shown, does not necessarily reflect protein levels and activities. In addition, a recent study revealed that 17*β*-HSD6 is expressed in ER*β*-positive epithelial cells of the human prostate but that in prostate cancers of Gleason grade higher than 3, both ER*β* and 17*β*HSD6 become undetectable [26], suggesting that formation of 3*β*-Adiol via 17*β*HSD6 from DHT could be considered an important growth regulatory pathway, but not a marker of prostate cancer aggressiveness [26]. Finally, future studies are ongoing to characterize the phase two metabolism of steroids derivatives that are understudied and could be a future target for castrate-resistant prostate cancer [27].

In summary, we developed an easy tool to profile individual intraprostatic enzymatic activity (steroid map or fingerprint) by characterizing conversion pathways in an intact tissue environment from fresh biopsies. Using this novel approach we found that 17*β*-HSD-isoenzymes and aromatase activity in prostate tissue cultures correlate with biological behaviour. This approach could be a novel useful tool for clinical monitoring of novel enzyme-targeting drugs.

## Figures and Tables

**Table 1:** Characteristics of enrolled subjects.

	BPH (*n* = 14)	PC (*n* = 12)
AGE		
Mean ± SD	67 ± 7	70 ± 7
(Range)	(56–79)	(68–84)

PSA	3.02 ± 1.58 ng/mL	9.13 ± 5.47 ng/mL

Gleason score	n.a.	Gleason < 8 (60%) Gleason ≥ 8 (40%)

Therapies	2/14 on *α*-lytic agents (no pts. on hormonal treatment)	No pts. on hormonal treatment

Additional notes	3/14 with chronic inflammation	
